# System-Wide Accelerated Implementation of Telemedicine in Response to COVID-19: Mixed Methods Evaluation

**DOI:** 10.2196/22146

**Published:** 2020-10-06

**Authors:** Diego Garcia-Huidobro, Solange Rivera, Sebastián Valderrama Chang, Paula Bravo, Daniel Capurro

**Affiliations:** 1 Department of Family Medicine Pontificia Universidad Catolica de Chile Santiago Chile; 2 Department of Internal Medicine Pontificia Universidad Catolica de Chile Santiago Chile; 3 Office of Innovation and Digital Transformation UC Christus Health Network Santiago Chile; 4 School of Computing and Information Systems University of Melbourne Melbourne Australia; 5 Centre for Digital Transformation of Health University of Melbourne Melbourne Australia

**Keywords:** telemedicine, telehealth, virtual medicine, health services evaluation, COVID-19

## Abstract

**Background:**

As the COVID-19 pandemic disrupted medical practice, telemedicine emerged as an alternative to outpatient visits. However, it is not known how patients and physicians responded to an accelerated implementation of this model of medical care.

**Objective:**

The aim of this study is to report the system-wide accelerated implementation of telemedicine, compare patient satisfaction between telemedicine and in-person visits, and report provider perceptions.

**Methods:**

This study was conducted at the UC Christus Health Network, a large private academic health network in Santiago, Chile. The satisfaction of patients receiving telemedicine care in March and April 2020 was compared to those receiving in-person care during the same period (concurrent control group) as well as in March and April 2019 (retrospective control group). Patient satisfaction with in-person care was measured using the Net Promoter Score (NPS) survey. Patient satisfaction with telemedicine was assessed with an online survey assessing similar domains. Providers rated their satisfaction and responded to open-ended questions assessing challenges, strategies used to address challenges, the diagnostic process, treatment, and the patient-provider relationship.

**Results:**

A total of 3962 patients receiving telemedicine, 1187 patients from the concurrent control group, and 1848 patients from the retrospective control group completed the surveys. Satisfaction was very high with both telemedicine and in-person services. Overall, 263 physicians from over 41 specialties responded to the survey. During telemedicine visits, most providers felt their clinical skills were challenged (61.8%). Female providers felt more challenged than male providers (70.7% versus 50.9%, *P*=.002). Surgeons, obstetricians, and gynecologists felt their clinical skills were challenged the least, compared to providers from nonsurgical specialties (*P*<.001). Challenges related to the delivery modality, diagnostic process, and patient-provider relationship differed by provider specialty (*P*=.046, *P*<.001, and *P*=.02, respectively).

**Conclusions:**

Telemedicine implemented in response to the COVID-19 pandemic produced high patient and provider satisfaction. Specialty groups perceived the impact of this new mode of clinical practice differently.

## Introduction

COVID-19 is the largest pandemic of the current century. It has generated transformative changes in health care, including the prioritization of emergency departments and intensive care units [1]. In addition, with physical distancing and isolation as major strategies to contain the spread of the disease, there are fewer patients receiving outpatient clinical care [2]. Although reducing in-person encounters is needed to contain the transmission of SARS-CoV-2, medical care is still needed for acute and chronic conditions.

In this context, telemedicine has emerged as a necessary clinical innovation to provide patient care [3]. This delivery modality has demonstrated clinical effectiveness, optimization in the delivery of health services, usability, and high patient and provider satisfaction [4,5]. Although appealing, several factors may hinder the sustainable use of telemedicine. First, providers prefer in-person encounters to virtual ones [6]. Potential concerns include uncertainty in the clinical encounter, difficulties performing a physical examination, and concerns with the patient-provider relationship [7]. These difficulties may reduce a clinician’s motivation to deliver virtual services. In addition, health insurance agencies have not always recognized this type of clinical service to be reimbursable, limiting the scaling up of telemedicine. Even though telemedicine has existed for several years and has had several implementation barriers, the COVID-19 pandemic has quickly transformed the practice of ambulatory medicine [8,9] and the willingness of health care organizations to implement this mode of delivery. The effect of rapidly deploying new models of care is still incompletely understood.

In this context, the UC Christus Health Network in Santiago, Chile, implemented telemedicine services two weeks after the first patient with COVID-19 was diagnosed in the country. The purpose of this paper is to report the system-wide accelerated implementation of telemedicine, compare patient satisfaction with telemedicine and in-person visits, and report provider perceptions regarding the implementation of virtual visits.

## Methods

### Design

This study used a convergent parallel mixed methods evaluation. In this study design, quantitative and qualitative methods have similar relevance, the data are collected in the same phase of the research project, the analysis of the data is independent, and the findings are combined and interpreted together [10]. We chose this design to better understand participants’ experiences. Quantitative data consisted of in-person and telemedicine visits, patient demographics, satisfaction, and challenges identified by clinicians. Data from patients receiving telemedicine care were compared to that of patients receiving in-person care between March 1 and April 30, 2019 (retrospective control group), and March 1 and April 30, 2020 (concurrent control group). Qualitative data included physicians’ responses to open-ended questions asking about challenges related to using telemedicine and mechanisms to address those challenges when delivering virtual health services. This study was approved by the Institutional Review Board of the Pontificia Universidad Católica de Chile.

### Setting

Chile’s health system combines public and private health providers. Public health services are paid for by public health insurance. Private health providers are paid per service delivered, and health insurance pays some or all of the fees charged by the health care providers.

This study was conducted at the UC Christus Health Network, a large private academic health network in Santiago, Chile. This health network has two hospitals with nearly 500 beds in total and 6 primary and secondary care clinics, with more than 800,000 patient visits every year. UC Christus has been nationally recognized for its innovation and patient service with the “Best Place to Innovate,” “Praxis Xperience Index,” and “ProCalidad” Awards.

### Telemedicine and its Implementation

UC Christus Health started implementing telemedicine on March 17, 2020, about two weeks after the first patient with COVID-19 was diagnosed in Chile. Initially, it started with generalist specialties, including internal medicine and family medicine, which offered services and guidance for patients with respiratory symptoms. Telemedicine was subsequently implemented as a strategy to provide care across the entire health care system. Currently, more than 720 providers from 61 clinical specialties are providing patient care through telemedicine.

To access these encounters, patients schedule appointments online or over the phone. When scheduling the appointment, patients must accept the terms of telemedicine, including that providers cannot provide certain services virtually, such as ordering restricted prescriptions or providing official health certificates. Once the appointment is scheduled, patients receive a confirmation email and are invited to pay for their visit. The cost of this service is about US $50, representing about two-thirds of the cost for in-person visits. Once the visit is paid for, patients and providers receive a Zoom link (Zoom Video Communications Inc) for their telemedicine visit, and they connect at the scheduled time. An administrative team supports patient-provider connections. Providers have access to the patient's electronic medical record and use a separate platform to order prescriptions, laboratory exams, images, and procedures, which are usually written manually during in-person visits. These electronic orders are emailed automatically to patients from the medication and laboratory platform.

### Outcomes and Data

#### Overview

Primary outcomes were patient and provider satisfaction along with providers’ challenges in implementing telemedicine. This study used four databases, including the following: (1) demographics of outpatient and telemedicine visits, (2) patient satisfaction with in-person visits, (3) patient satisfaction with telemedicine visits, and (4) provider satisfaction with telemedicine visits. Demographic data were extracted from patients’ electronic medical records for the complete months of March and April of 2019 and 2020, generating three different groups: 2019 in-person care (retrospective control group), 2020 in-person care (concurrent control group), and 2020 virtual care (telemedicine group). Satisfaction data from patients and providers were stored in independent databases.

#### Patient Demographics

The number of visits and physician’s specialty, as well as patient’s sex, date of birth, insurance type, and address for all visits were extracted from electronic medical records. In this study, physician specialty was classified as one of the following seven groups: generalist specialties (family medicine, general internal medicine, geriatrics, pediatrics), pediatric subspecialties (including genetics and infant neurology), internal medicine subspecialties (including neurology), surgical subspecialties (including anesthesia, orthopedics, urology), obstetrics and gynecology, psychiatry, and ophthalmology, dermatology, and otolaryngology.

#### Patient Satisfaction With In-Person Visits

Patient satisfaction is usually evaluated using the Net Promoter Score (NPS) survey (Cronbach α=.96) (FBA Consulting, unpublished material, 2020) [11]. This measure assesses satisfaction across several dimensions including access, payment process, infrastructure, and provider’s services, as well as general satisfaction, using a 5-point Likert scale (1=very bad, 5=very good). Data were collected through an online survey sent via SMS text messaging after the visit.

#### Patient Satisfaction With Telemedicine Visits

An 8-item questionnaire was developed to assess patient satisfaction with telemedicine services using a 1-7 scoring system. Like the survey completed by patients receiving in-person services, survey domains included questions assessing satisfaction with access, payment process, web portal (infrastructure), and provider’s services, as well as general satisfaction (Cronbach α=.86). This evaluation was sent via email after the encounter.

#### Physician Satisfaction With Telemedicine

Using a 19-item anonymous survey, clinicians were asked about their demographics, telemedicine experience, and general satisfaction. Open-ended questions assessed challenges in care delivery, diagnostic process, treatment, and the patient-provider relationship, as well as strategies used to overcome these barriers.

#### Data Analyses

Following the procedures of the selected mixed methods design, each type of data was analyzed independently.

#### Quantitative Data

Patient and provider demographic information was summarized using descriptive statistics. Trends for in-person and telemedicine visits were plotted and compared to the null hypothesis of no trend using the chi-square test for trend. Patient characteristics of those receiving telemedicine services were compared to the demographics of patients from the retrospective and concurrent control groups using the chi-square test. As satisfaction data did not have a normal distribution, the Wilcoxon-Mann-Whitney test was used, after converting in-person and telemedicine scoring systems to a 0-100 scale. Responses regarding considering telemedicine as challenging and counts of qualitative responses were modeled after conducting bivariate analysis with logistic regressions that included the physician’s specialty category as an independent variable, and gender, age, years of clinical experience, and telemedicine experience as covariables. Adjusted odds ratios, predicted probabilities, and 95% confidence intervals for the selected outcomes were estimated for each specialty category. All analyses were conducted in STATA (Version 14; StataCorp) and resulting *P* values <.05 were considered statistically significant.

#### Qualitative Data

Responses to open-ended questions were coded by two independent researchers following the procedures of content analysis [12]. Using a combination of inductive and deductive approaches, physicians’ perceptions were grouped into emerging categories and subcategories organized according to the domains of the guiding questions. To ensure coding reliability, a random sample of 20% of the clinician surveys (n=53) were double-coded, resulting in 96.4% coding agreement. Responses in each category were counted and grouped by domain. Quotes were selected to represent the participant’s opinions and these were translated to English.

Mixed methods integration was conducted at the finding interpretation and reporting phases. To interpret both sources of data jointly, researchers gathered to discuss quantitative and qualitative results together. Integration at the reporting level occurred through a continuous narrative approach, in which the mixed data are presented in a single report, but in different sections [13].

## Results

### Change in Clinical Practice

**Figure 1:**
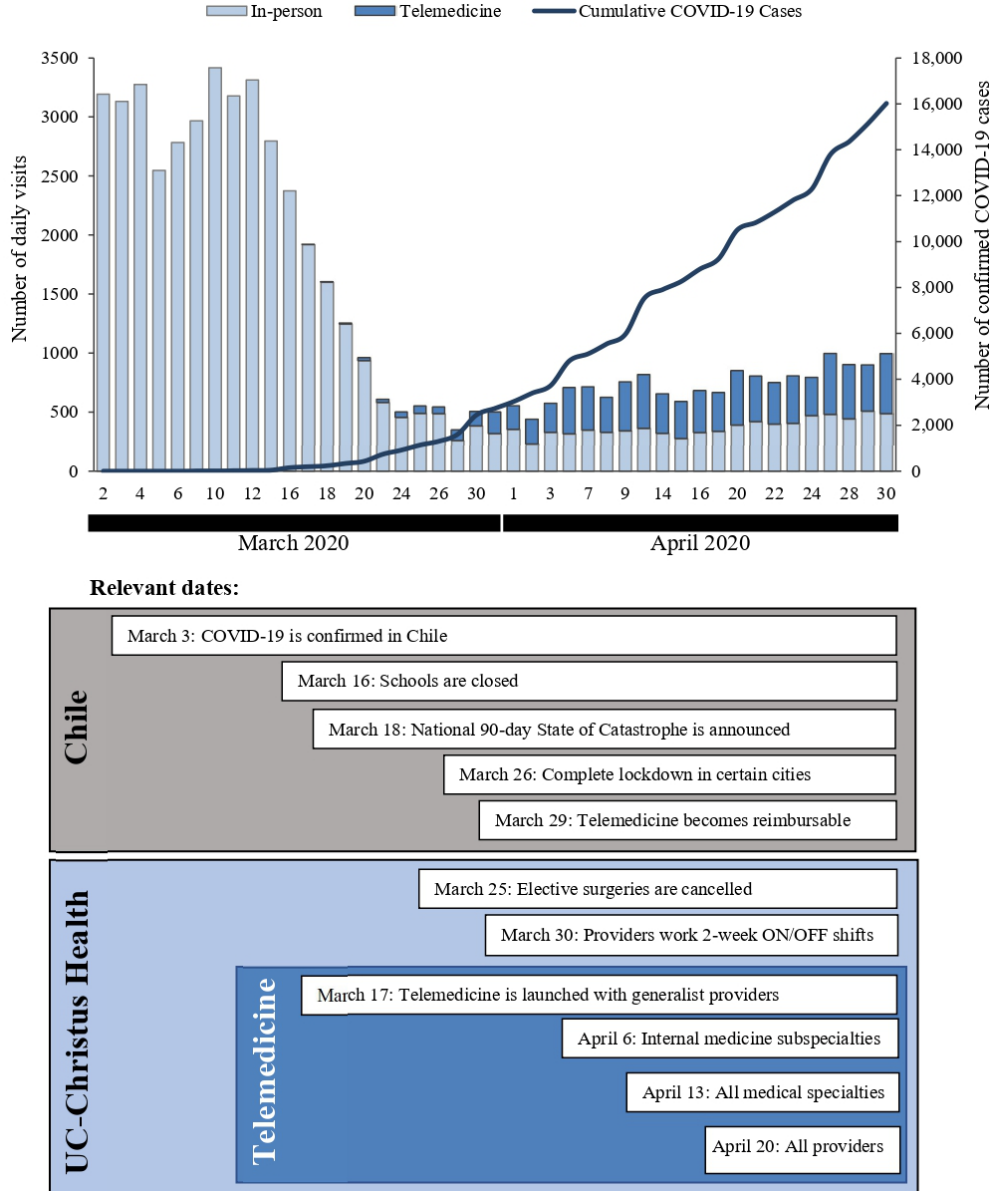
Rates of in-person and telemedicine visits at UC Christus Health and cumulative COVID-19 cases from March 1 to April 30, 2020, as well as relevant dates of COVID-19 management in Chile and UC Christus Health.

In 2019 and 2020 before COVID-19 arrived in Chile, there were an average of 3039 and 3163 daily outpatient visits in the UC Christus Health Network, respectively. In-person visits were reduced to an average of 384.7 daily visits 3 weeks after the first patient with COVID-19 was confirmed in Chile, representing an 87.9% reduction in outpatient visits (Figure 1, *P*<.001). Since telemedicine services began, the number of daily visits has increased, with the UC Christus Health Network delivering up to 509 virtual visits each day (*P*<.001).

**Figure 2:**
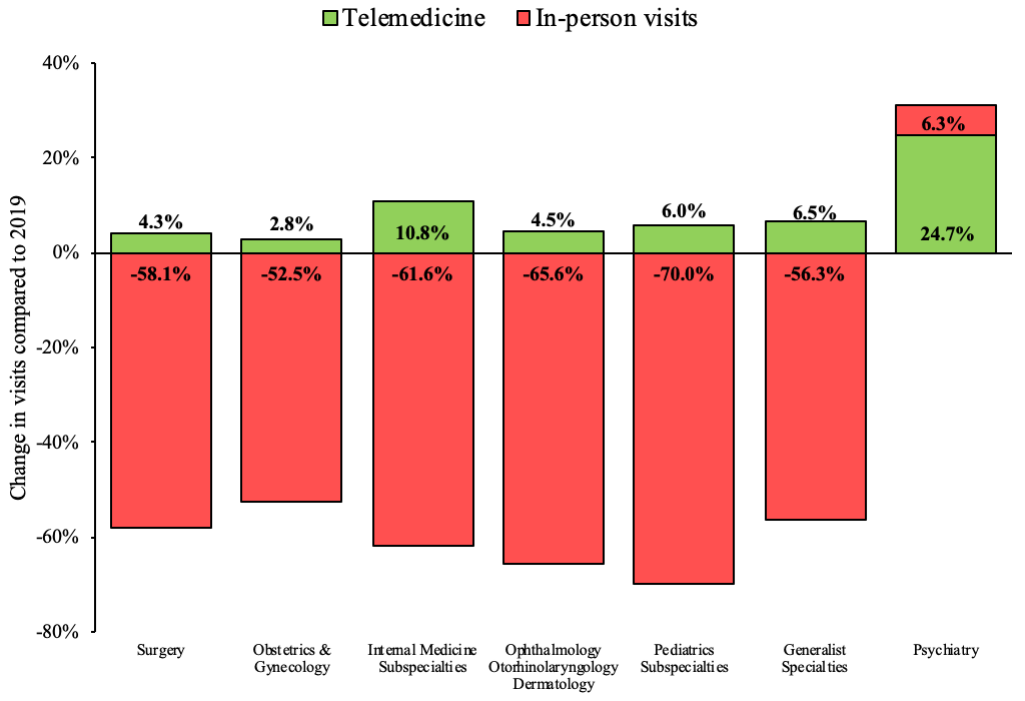
Rates of telemedicine and in-person visits by medical specialty.

Figure 2 displays telemedicine and in-person activity across medical specialties during 2020 compared to 2019. All specialties except psychiatry decreased the number of in-person visits. Pediatrics subspecialties, along with ophthalmology, otorhinolaryngology, and dermatology had the greatest reduction of in-person visits, experiencing between 61.6% to 70.0% of the demand of the previous year (*P*<.001).

Physicians from all specialty groups were able to implement virtual visits. Even though psychiatrists have been able to provide up to 24.7% of their 2019 volume of clinical encounters through virtual care, most medical groups have only delivered between 2.8% and 10.8% of visits compared to their 2019 baseline (*P*<.001).

### Patient Demographics

Table 1 compares the demographic characteristics of the telemedicine, concurrent control, and retrospective control groups. Patients accessing telemedicine were more likely to be female and have private insurance, and less likely to be children or older adults, or residents of the Santiago Metropolitan Region compared to both in-person control groups (*P*<.01 for all comparisons).

**Table 1:** Demographic characteristics of patients receiving care through telemedicine compared to patients from concurrent and retrospective control groups.

Characteristics		Telemedicine group (n=8592), n (%)	Concurrent control group (n=51,290), n (%)	*P* value	Retrospective control group (n=127,669), n (%)	*P* value
Gender (female), n (%)		5376 (62.6)	31,436 (61.4)	.024	77,845 (61.0)	.003
**Age category (years), n (%)**		N/A^a^	N/A	<.001	N/A	<.001
	0-18	1749 (20.4)	11,855 (23.1)	N/A	34,357 (26.9)	N/A
	19-64	5725 (66.6)	31,607 (61.6)	N/A	73,632 (57.7)	N/A
	≥65	1118 (13.0)	7828 (15.3)	N/A	19,680 (15.4)	N/A
Private health insurance, n (%)		7542 (87.8)	21,319 (41.6)	<.001	56,495 (44.3)	<.001
Resident of the Santiago Metropolitan Region, n (%)		6529 (76.0)	42,833 (83.5)	<.001	111,822 (87.6)	<.001

^a^N/A: not applicable.

### Patient Satisfaction

**Figure 3:**
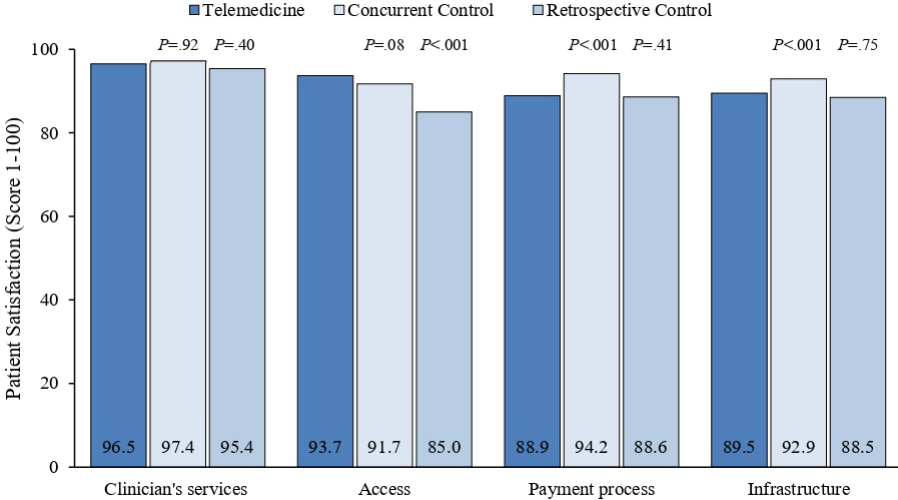
Satisfaction of patients receiving care via telemedicine compared to concurrent and retrospective control groups.

During March and April of 2019 and 2020, 1848 and 1187 patients responded to the satisfaction questionnaire after the in-person visits, respectively. In addition, 3962 patients responded to the telemedicine satisfaction assessment. Satisfaction was very high with both telemedicine and in-person services (Figure 3). Patients receiving telemedicine services reported similar satisfaction with clinician’s services compared to both control groups. Patients using telemedicine reported similar access to providers compared to in-person visits in 2020 (*P*=.08), but greater access compared to in-person visits in 2019 (8.7% increase, *P*<.001). Patients using telemedicine care reported less satisfaction with the payment process (5.3% reduction, *P*<.001) and infrastructure (web portal, 3.4% reduction, *P*<.001) compared to patients receiving in-person visits concurrently. Ratings in these domains were not statistically different compared to patients receiving in-person visits in 2019.

### Physician Satisfaction

A total of 263 clinicians responded to the satisfaction assessment (36.5%). They were mostly female (n=155, 58.9%), with an average age of 44 years (SD 10.9), and an average of 16.8 years (SD 11.4) of clinical experience. Most providers reported limited experience with telemedicine, with 147 clinicians (61%) reporting 10 or fewer virtual patients encounters at the time they responded to the questionnaire. There were no differences in gender (*P*=.37), age (*P*=.10), or specialty category (*P*=.32) among survey respondents and nonrespondents (n=721 providers). Overall, 244 providers were satisfied or very satisfied with telemedicine (92.8%), and most would recommend this service to friends or family members (94.2%).

### Experiencing Challenges When Conducting Telemedicine Visits

**Figure 4:**
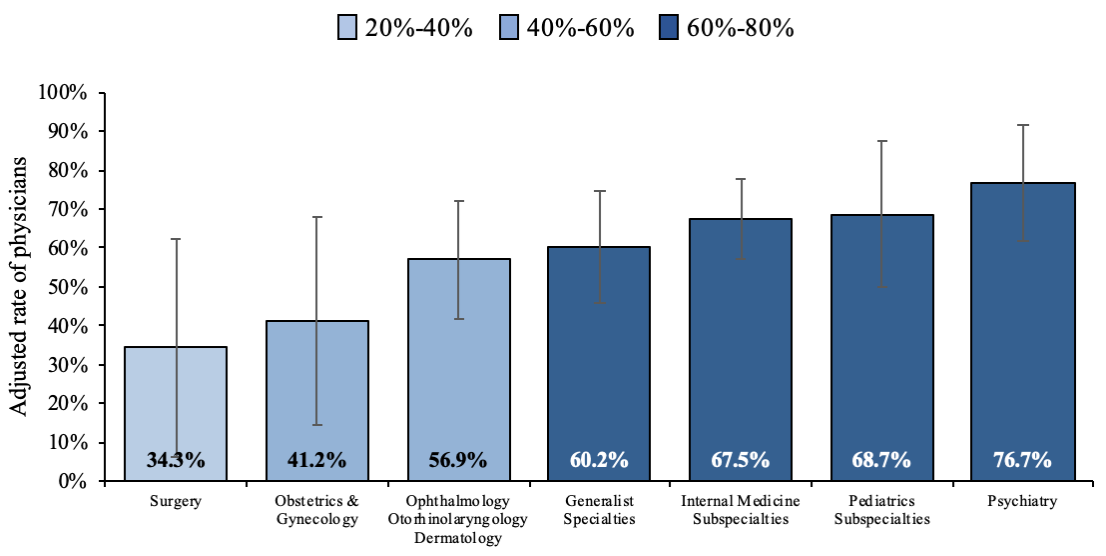
Adjusted rate and 95% CIs of physicians perceiving their clinical skills being challenged when using telemedicine, by physician’s specialty.

When providing telemedicine care, most physicians felt their clinical skills challenged somewhat or a lot (61.8%). Female providers felt more challenged than male providers (70.7% versus 50.9%, *P*=.002). Surgeons, obstetricians, and gynecologists felt that their clinical skills were challenged the least, compared to providers from other medical specialties (*P*=.02). These associations persisted after adjusting for covariates in a logistic regression model (Figure 4). There were no statistically significant differences in feeling that clinical skills were challenged according to the provider’s age, years of clinical experience, or the number of patients seen virtually.

### Specific Challenges With Telemedicine and Mechanisms of Addressing Them

Table 1 in Multimedia Appendix 1 summarizes the categories and subcategories of challenges experienced with telemedicine delivery, the diagnostic process, and the patient-provider relationship. Most challenges were related to the delivery mode, specifically accessing the platforms used, dealing with patient scheduling, and accessing the resources required for telemedicine. There were no challenges specifically related to patient treatment. Several strategies were implemented by physicians to address these barriers (Table 2 in Multimedia Appendix 1). Strategies most commonly used were directly contacting the patient, and requesting support from the help center, peers, or patients.

**Figure 5:**
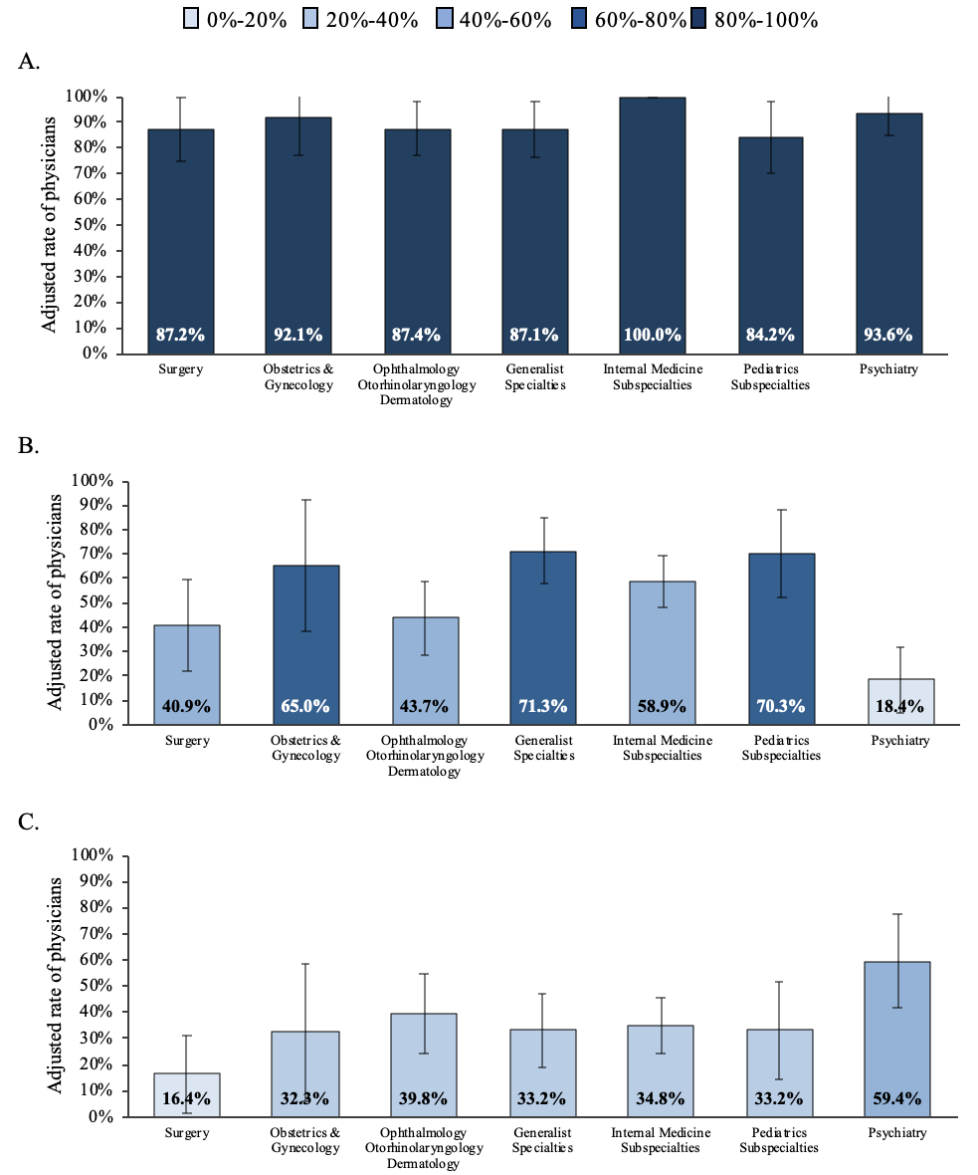
Adjusted rate and 95% CIs of providers reporting challenges with (A) the modality of service, (B) the patient’s diagnostic process, and (C) the patient-provider relationship, by physician’s specialty.

Figure 5 represents the rates of physicians experiencing any challenge with the delivery modality, diagnostic process, and patient-provider relationship according to their specialty category. Providers from all specialties experienced challenges with the delivery system, especially providers from internal medicine subspecialties and psychiatry (Figure 5A, *P*=.046). There were differences by specialty when reporting challenges related to a patient’s diagnostic process. Most physicians who experienced challenges were from primary care and pediatrics subspecialties (Figure 5B, *P*<.001). Challenges in the patient-provider relationship also differed by specialty group. About 60% of psychiatrists reported experiencing challenges in this domain compared to 16.4% of surgeons (Figure 5C, *P*=.02).

## Discussion

### Main Results

The COVID-19 pandemic has radically transformed clinical care and our organization was forced to quickly deploy telemedicine services without the standard change management and training strategies that are usually required for successful digital transformations in health care [14]. Despite this, we have seen a quick uptake and the practice has been widely accepted in a short period of time [15]. According to Kotter’s change management process, the first step toward successful change is the sense of urgency to change [16]; it is undeniable that the COVID-19 pandemic has generated an enormous sense of urgency to adapt to this reality. In a few weeks, we were able to include all clinical specialties in the telemedicine project. Moreover, the Chilean National Health Fund (FONASA), which had delayed including clinical specialties in funding schemes for years, established insurance coverage for telemedical services three weeks after the first COVID-19 case in Chile [17]. The COVID-19 pandemic created the perfect environment for providers and health care organizations to bring down long-lasting barriers to the implementation of telemedicine and other digital innovations [18].

### Comparison With Prior Work

The fear of becoming infected with COVID-19 in health care settings drove many patients away from in-person care [19]. Specialties that focus on children or require medical equipment (eg, otorhinolaryngology or ophthalmology) experienced the highest reduction of in-person visits. This is consistent with recent studies that identified a lack of access to laryngoscopes as a downside of telemedicine visits [20]. Even though telemedicine has provided physicians with an option to deliver outpatient services, the recovery of outpatient clinical activity levels toward 2019 levels differed between specialty groups. Psychiatrists and internal medicine subspecialists have been able to implement the highest number of virtual visits. This different level of adoption could be attributed to patient or clinician characteristics and could be further explored in future studies. In addition, differences were observed in the demographics of patients accessing telemedicine services compared to in-person visits. To continue the growth of this modality of care among all medical groups, and reduce disparities in health care access, further innovations should be developed to provide access to the patient and provider groups that have unequally benefited from this clinical transformation. For example, physicians could use patient-controlled devices to supply equipment normally available in offices [21], and marketing can be focused on groups with less access to care. It is noteworthy that telemedicine increased access to specialty care that previously could have been limited by geographic barriers [22].

Overall, both clinicians and patients were highly satisfied with the implemented telemedicine services. This is partially comparable to the findings from a previous systematic review that synthesized 17 studies assessing patient and physician perceptions of telemedicine versus in-person consultations in different medical specialties [23]. The systematic review found that most studies reported no significant differences in patient-related outcomes such as convenience, format of the consultation, or rapport. In addition, the same systematic review found that overall, physicians showed reduced satisfaction with telemedicine visits regarding communication (patient-provider relationship), and physical examination or diagnostic assessment. These findings are compatible with the results of our study, as some of the concerns identified by providers related to the impact of telemedicine on diagnostic assessment and the patient-provider relationship, as well as complications associated with the implemented delivery modality. Even though the system was far from perfect (eg, using three different platforms during a virtual encounter), patients and providers valued telemedicine as an innovation that mitigated the risk of infection associated with in-person visits. Reducing the risk of contagion has been identified as a likely facilitator to adopting telemedicine [24].

In parallel with a high overall satisfaction with telemedicine, we observed that more than 60% of all clinicians felt that their clinical skills were challenged during virtual visits. This is similar to a previous study that reported an increase in overall workload, mental effort, and psychological stress during virtual visits [25]. We also observed differences across specialties in the perception of telemedicine consultations and how telemedicine can challenge their clinical skills; those in technical specialties found telemedicine less challenging than those in relationship-focused specialties [26,27]. This difference in the physicians’ orientation toward patient care might also reflect differences in adjusting to a new service delivery process.

When analyzing the types of challenges faced by clinicians and the strategies used to address them, it is noticeable that most of the challenges faced were due to the delivery mode, the platform used, and patient scheduling. This finding was identified by embedding a qualitative assessment to the quantitative survey and is consistent with a Cochrane systematic review that found that technical difficulties experienced by clinicians during telemedicine services produced high dropout rates [28]. As telemedicine was quickly deployed as a system-wide innovation in response to COVID-19, there was no time to produce a user-friendly platform, but this will be fundamental to maintaining clinician engagement with virtual services as the pandemic evolves as well as once the pandemic is over. In addition, for telemedicine to overcome all identified challenges, further training is needed among providers. Although medical schools have embraced online training [29], medical students should also be trained in telemedicine [30]. The presented system-wide implementation required that all physicians, regardless of their specialty, were knowledgeable about virtual care. As providers become more experienced conducting telemedicine encounters, it is likely that these barriers will be reduced over time and their experience should be considered as valuable input for future curriculum design.

In the patient-clinician relationship domain there were striking differences between specialty groups. This is especially relevant since psychiatrists experienced the greatest relative increase in telemedicine consultations. We did not explicitly seek to study the causes of this difference, but it is probably a consequence of the differing types of clinician-patient relationships between specialties [26,27]. This is relevant since mental health is one of the areas that has seen the greatest increase in uptake in the past years [31] and it has been a major concern during the COVID-19 pandemic [32]. Mental health telemedicine services are, on average, equally effective when compared to face-to-face consultations [4,28].

### Limitations

Although the implementation of virtual care in response to COVID-19 has been reported in different settings [24,33-38], this is the first report of a system-wide accelerated implementation of telemedicine services using quantitative and qualitative methods. This large-scale implementation allowed us to identify and compare visits and challenges experienced by different specialty groups. However, as any other evaluation, this paper has limitations that are important to acknowledge. First, although there were no differences in the demographics and medical characteristics of providers who did and did not respond to the satisfaction survey, selection bias is always present in voluntary surveys [39]. This limitation also affects patient satisfaction ratings. In this group, because the available data consisted of anonymous ratings, we could not assess potential responder bias. Second, because of the rapid deployment of telemedicine services, we developed a patient satisfaction questionnaire to assess patient satisfaction with telemedicine similar to the NPS but could have used validated measures [40] and integrated qualitative components, as we did in the developed questionnaire for providers. Finally, COVID-19 not only affected health care provider priorities but also changed patient priorities. For example, during the pandemic, patients may be less likely to seek care for chronic conditions, and this could affect patient satisfaction. Future system-wide evaluations of telemedicine should be conducted once the current pandemic is controlled. Despite these limitations, this report highlights the challenges related to implementing telemedicine experienced by multiple groups of physicians and the mechanisms they used to address these challenges during the COVID-19 pandemic.

### Conclusions

Telemedicine produced high satisfaction among patients and providers. Although this modality of clinical care was rapidly deployed in response to the COVID-19 pandemic, there was high heterogeneity in its implementation across medical specialties. These differences need to be considered in future implementations of telemedicine when the current medical context is addressed.

## Appendix

Multimedia Appendix 1 Supplementary tables.

## Abbreviations

NPS: Net Promoter Score
