# Supplementary material for: System-Wide Accelerated Implementation of Telemedicine in Response to COVID-19: Mixed Methods Evaluation
Source: J Med Internet Res. 2020 Oct 6;22(10):e22146. doi: 10.2196/22146 (PMC7541041; doi:10.2196/22146)
Supplement: Multimedia Appendix 1 [file jmir_v22i10e22146_app1.docx]

eTable 1: Categories, subcategories and selected quotes of challenges experienced implementing telemedicine according to the modality of service, diagnostic process, and patient-provider domains

| **Domain** | **Category** | **Frequency (%)** | **Sub-categories** | **Selected quotes** |
| --- | --- | --- | --- | --- |
| **Modality of Service** | Access to the electronic health record, order software, and ZOOM | 175 (66.5%) | - Access to the electronic health record - Platforms are not user-friendly - Difficulty finding lab orders or prescriptions - Ability to see lab results done in other health organizations - Connecting to ZOOM audio - Stealing passwords | - *“The clinical record is the weakest point. When using a Mac it’s not easy and to use”* - *“Problems with ZOOM. Once I could not activate the audio and ended up completing the visit over the phone”* |
|  | Patient scheduling | 119 (45.3%) | - No show - Last minute scheduling - Scheduling mistakes (specialty or age group) - Few patients - Foreign patients - Scheduling patients with severe conditions (e.g., suicide attempts, severe respiratory distress) - Allotted time is insufficient for a comprehensive evaluation - Scheduling patients that require in-person care | - *“Many patients schedule a visit but never pay on time. To be able to schedule they should pay directly so those appointments are not lost”* - *“Some patients were not from my specialty”* |
|  | Resources for telemedicine | 102 (38.8%) | - Type of personal computer - Patient and provider internet access - Incapacity to order ‘restricted prescriptions’ - Incapacity to provide official forms or certificates - Inability to print a visit summary | - *“Sometimes the internet blinks and I need to log in again and finish writing in the electronic health record”* - *“Impossible to write a ‘restricted prescription’”* |
|  | Patients lack clarity of the service | 20  (7.6%) | - Lack of consent - Cost - Insurance reimbursement - Limitations of the modality of care (e.g., ‘restricted prescriptions’ or official certificates’ | - *“In my experience, no patients have received (or read) any ‘disclaimer’ or ‘consent’ about the limitations of the virtual visits”* |
|  | Lack of training | 5  (1.9%) | - Lack of training in the programs used | - *“The challenges were confronting a new modality of care without an adequate training”* |
| **Diagnostic Process** | Lack of physical examination | 134 (51.0%) | - Lack of physical examination - Inability to do in-person tests through the screen (e.g., cognitive evaluation) | - *“In the cases that ask for skin lesions it is important to have patient´s pictures. The camera has very little resolution and many visits are at night with little light”* |
|  | High uncertainty | 10 (3.8%) |  | - *“Not examining a patient produces uncertainty, but patients are understanding and supportive of the self-exam”* |
| **Patient-Provider Relationship** | Development of a close relationship | 45 (17.1%) | - Difficulty stablishing a warm relationship - Supporting patients during the visit | - *“I have a lot of patience and do education. Before I begin the visit, we talk and try to reassure patients. That’s what I do the most”* |
|  | Providing continuity of care | 29 (11.0%) | - Email follow-up - Patients do not schedule a follow-up visit | - *“To open the possibility to remain connected through any contingency. This way the patient is more satisfied and has the feeling of continuity, not just a casual contact”* |
|  | Technical issues negatively affecting the relationship | 19  (7.2%) |  | - *“Maintaining a warm environment regardless of the audio or video cuts that a call might have”* |
|  | Ensuring privacy | 8  (3.0%) | - Providing personal email or phone number - Ensuring that the patient’s location is private - Patients don’t feel comfortable | - *“There are patients that prefer an in-person visit. It is hard with small children, and for adolescents sometimes privacy at home is lacking”* |
|  | Provide care for certain groups of patients (e.g., children, elders) | 6  (2.3%) | - Providing care for young children - Difficulty communicating with elders | - *“To evaluate pediatric patients that don’t cooperate like infants”* |
|  | Patient communication | 6  (2.3%) | - Difficulty confirming patient’s understanding - Difficulty assessing non-verbal language | - *It has to do with the capacity to contain a patient when needed. The traditional skills of a personal encounter are lost, and this might be principal challenge of this modality of care”* |
|  | Patient honesty | 4  (1.5%) | - Lack of patient honesty - Patient scheduled a visit under another person’s name | - *“A mother wanted a prescription for fluoxetine and was lying saying that her daughter used it before”* |
|  | Involving other family members | 3  (1.1%) |  | - *“It is hard to talk to all family members in a virtual visit”* |

eTable 2: Categories, subcategories and selected quotes of addressing mechanisms implementing telemedicine according to the modality of service, diagnostic process, and patient-provider domains

| **Domain** | **Category** | **Frequency (%)** | **Sub-categories** | **Selected quotes** |
| --- | --- | --- | --- | --- |
| **Modality of Service** | Contact personally the patient through email, text message or phone call | 127 (48.3%) | - Calling patients - Sending a text message - Sending an email | - *“In case of problems with ZOOM, I have used a phone call or WhatsApp”* - *“When I don´t find a prescription, I send an email with a picture of a hand-written prescription”* |
|  | Request technical support | 107 (40.7%) | - Support from support center - Support from peers - Support from patients | - *“Patient connection problems have been able to be solved by the support center”* |
|  | Create an in-person support system | 50 (19.0%) | - Refer to an in-person visit - Request an in-person pick up of a restricted prescription or official certificate - Sending a restricted prescription through delivery systems | - *“Referring patients for in-person visits”* - *“I have sent ´restricted prescriptions´ using Uber* - *“Colleagues that are providing in-person services have given official certificates to my patients”* |
|  | Receive training and practice | 50 (19.0%) | - Receiving online training - Practicing with patients, friends or family members | - *“With patience and practice”* - *“I searched for an online training in telemedicine”* |
|  | Explain to the patient about the scope and limitations of telemedicine | 34 (12.9%) |  | - *“Explaining the limitations of the virtual care and being creative about the solutions”* |
|  | Take longer time with the patient | 33 (12.5%) | - Conduct a longer visit - Complete the electronic health record after the visit | - *“Giving much more time to the visit, leaving time for questions”* |
|  | Support the patient directly through ZOOM | 20  (7.6%) | - Chatting - Sharing screens - Modifying the lab order form - Modifying the prescription form | - *“With the platform problems, I use the chat to give instructions, and when impossible, I give patients a call. I have used the ´screen sharing option´ to explain”* |
|  | Obtain additional support systems | 16  (6.1%) | - Conduct visit using different platforms (e.g., Facetime, Skype, Google Meet) - Use cell phone´s internet - Use more than a computer - Purchase additional software | - *“With a patient that their audio did not work, I called him through ´Facetime´ and solved the problem”* |
|  | Return payment | 1  (0.4%) |  | - *“There was no other option than giving them the money paid for the visit back”* |
| **Diagnostic Process** | Ask for patient’s support | 58 (22.1%) | - Request pictures - Request self-examination - Request moving patient´s device camera - Use patient´s medical equipment (e.g., thermometer, sphygmomanometer, etc.) | - *“I have requested the patient to move the illumination and the camera to observe the oral cavity”* |
|  | Take an exhaustive history | 48 (18.3%) |  | - *“Taking an exhaustive history, giving it more time when the case seems to be more complex”* |
|  | Request additional labs, images or procedures | 23  (8.8%) |  | - *“I have had to request more lab tests to have more certainty”* |
|  | Fully review the electronic medical record | 9  (3.4%) |  | - *“I review the electronic medical record before the visits”* |
|  | Ask for patient’s family member support | 7  (2.8%) |  | - *“I have asked family members to do a physical examination to patients”* |
| **Patient-Provider Relationship** | Intensify the use of communication skills | 61 (23.2%) | - Simplifying instructions - Provide written instructions - Creating a positive patient-provider relationship - Using open-ended questions - Summarizing the visit - Putting attention to non-verbal communication | - *“Reflect a lot and checking if I am understanding correctly. Be very aware of the non-verbal language of the patient and mine”* |
|  | Be nicer | 16  (6.1%) |  | - *“Trying to be nicer and most empathic possible”* - *“Maintaining a positive relationship and asking for questions”* |
|  | Involve family members | 9  (3.4%) |  | - *“I have invited family members to join the conversation, bring their concerns to solve them during the visit”* |
